# The Influence of Bacteriophages on the Metabolic Condition of Human Fibroblasts in Light of the Safety of Phage Therapy in Staphylococcal Skin Infections

**DOI:** 10.3390/ijms24065961

**Published:** 2023-03-22

**Authors:** Katarzyna Kosznik-Kwaśnicka, Małgorzata Stasiłojć, Grzegorz Stasiłojć, Natalia Kaźmierczak, Lidia Piechowicz

**Affiliations:** 1 Department of Medical Microbiology, Faculty of Medicine, Medical University of Gdańsk, Dębowa 25, 80-204 Gdansk, Poland; 2 Department of Cell Biology and Immunology, Intercollegiate Faculty of Biotechnology of University of Gdańsk and Medical University of Gdańsk, Dębinki 1, 80-211 Gdańsk, Poland

**Keywords:** phage therapy, MDRSA, MRSA, *Staphylococcus aureus*, bacteriophages, fibroblasts, neutral red, BJ

## Abstract

Phage therapy has been successfully used as an experimental therapy in the treatment of multidrug-resistant strains of *Staphylococcus aureus* (MDRSA)-caused skin infections and is seen as the most promising alternative to antibiotics. However, in recent years a number of reports indicating that phages can interact with eukaryotic cells emerged. Therefore, there is a need to re-evaluate phage therapy in light of safety. It is important to analyze not only the cytotoxicity of phages alone but also the impact their lytic activity against bacteria may have on human cells. As progeny virions rupture the cell wall, lipoteichoic acids are released in high quantities. It has been shown that they act as inflammatory agents and their presence could lead to the worsening of the patient’s condition and influence their recovery. In our work, we have tested if the treatment of normal human fibroblasts with staphylococcal phages will influence the metabolic state of the cell and the integrity of cell membranes. We have also analyzed the effectiveness of bacteriophages in reducing the number of MDRSA attached to human fibroblasts and the influence of the lytic activity of phages on cell viability. We observed that, out of three tested anti-*Staphylococcal* phages—vB_SauM-A, vB_SauM-C and vB_SauM-D—high concentrations (10^9^ PFU/mL) of two, vB_SauM-A and vB_SauM-D, showed a negative impact on the viability of human fibroblasts. However, a dose of 10^7^ PFU/mL had no effect on the metabolic activity or membrane integrity of the cells. We also observed that the addition of phages alleviated the negative effect of the MDRSA infection on fibroblasts’ viability, as phages were able to effectively reduce the number of bacteria in the co-culture. We believe that these results will contribute to a better understanding of the influence of phage therapy on human cells and encourage even more studies on this topic.

## 1. Introduction

New alternative therapeutics are being developed in response to the increase of antibiotic resistance among bacteria. Bacteriophages, the viruses that infect bacteria, are seen as one of the main candidates to replace antibiotics in the treatment of bacterial infections. For a long period of time, it was believed that phages do not interact with eukaryotic cells and therefore are neutral to higher vertebrates. However, recent studies have provided clear evidence that bacteriophages do interact with eukaryotic cells, can penetrate through membranes and interact with the immune system of humans or animals [1,2,3]. Barr et al., and Bichet et al., have demonstrated that phages are able to enter the inside of cells and penetrate through cell layers, though the intensity of these processes seems to depend on the phage as well as the type of the eukaryotic cell [1,4,5]. These observations have been corroborated by observations made in vivo in patients receiving phage therapy [2,6,7]. Therefore it is crucial to fully understand the effects that phages can have on eukaryotic cells in order to assess the safety of phage therapy before it can be applied as standard treatment [6,8].

*Staphylococcus aureus* is a pathogen that causes various types of infections and is the most common bacterium responsible for purulent infections of the skin and soft tissue [9]. In recent years the number of infections caused by multidrug-resistant strains of *S. aureus* (MDRSA) is rapidly increasing in hospitals around the world, often outnumbering the infections caused by antibiotic-susceptible strains [10,11]. Furthermore, there are more reports of skin infections caused by MDRSA outside of the hospital setting [12]. Therefore, phage therapy is seen as one of the leading alternatives to antibiotic therapy in order to treat MDRSA-caused skin and soft tissue infections, especially since many successful case studies have been reported [13]. However, the safety of the treatment is still to be analyzed in various research models as new information on phage-cell interactions comes into light [3,5,14]. Furthermore, as phages are applied at the site of infection, they would multiply as a result of their life cycle inside their bacterial host. This would result in cell lysis, the release of lipoteichoic acids from the bacterial cell wall and an increase in inflammation and apoptosis leading to possible side effects of therapy and worsening of the patient’s condition [15,16]. Therefore, it should be crucial to examine the influence of phage multiplication and bacterial lysis on eukaryotic cell viability [3,17]. In our work, we have decided to analyze the metabolic condition of normal human fibroblasts when exposed to different concentrations of three staphylococcal bacteriophages that have proven activity against clinical strains of MDRSA, vB_SauM-A, vB_SauM-C and vB_SauM-D, in order to assess their safety as potential antibacterial agents against *S. aureus*-caused skin infections [18,19]. We have also assessed the lytic activity of those phages in an MDRSA-infected fibroblast cell culture and analyzed the cell viability during the treatment in order to establish whether bacterial lysis could have a potentially harmful effect on human cells, as there is little research discussing what happens to eukaryotic cells in response to high levels of lipoteichoic acids and bacterial DNA that suddenly appear in the environment as a result of bacterial lysis by bacteriophages [3,6]. The aim of this study was therefore to assess whether phages vB_SauM-A, vB_SauM-C and vB_SauM-D influence the viability of human fibroblasts, if they could effectively reduce the number of bacteria attached to cell surfaces and if their lytic activity results in inhibition or increase of toxicity caused by bacterial presence.

## 2. Results

### 2.1. The Metabolic State of BJ Cells Treated with Phages

In order to analyze if phages vB_SauM-A, vB_SauM-C and vB_SauM-D could influence the viability of human fibroblasts, three concentrations of phages (10^9^, 10^8^, 10^7^ PFU/mL) were analyzed using the neutral red uptake and ATP level analysis.

**Figure 1:**
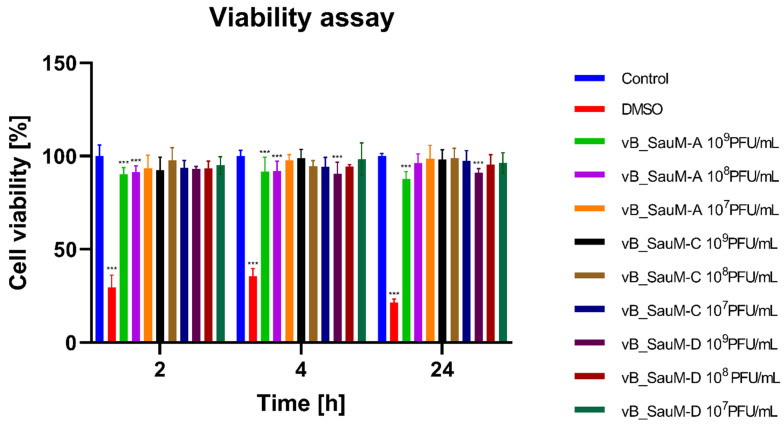
Cell viability of the BJ cells treated with phages vB_SauM-A, vB_SauM-C and vB_SauM-D at different concentrations after 2, 4 and 24 h of incubation in comparison to the untreated negative control (blue) and 10% DMSO-treated positive control (red). Mean values from three independent experiments are shown, with error bars representing SD. Statistical analysis was performed using the Kruskal–Wallis test followed by Dunn’s multiple comparison test for values with nonparametric distribution, with *** *p* < 0.001.

**Figure 2:**
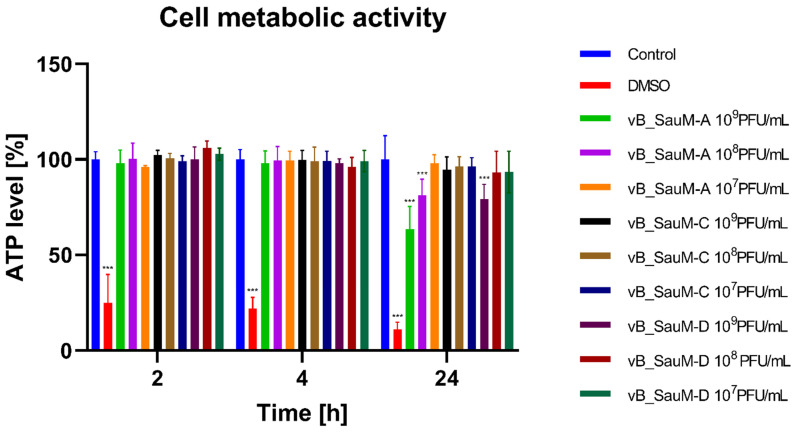
ATP levels of BJ cells treated with phages vB_SauM-A, vB_SauM-C and vB_SauM-D at different concentrations after 2, 4 and 24 h of incubation in comparison to the untreated negative control (blue) and 10% DMSO-treated positive control (red). Mean values from three independent experiments are shown, with error bars representing SD. Statistical analysis was performed using the Kruskal–Wallis test followed by Dunn’s multiple comparison test for values with nonparametric distribution, with *** *p* < 0.001.

We observed that high concentrations of phage vB_SauM-A (10^9^ and 10^8^ PFU/mL) caused a decrease in viability measured by neutral red uptake by 8–10%, which was determined as statistically significant (Figure 1). Additionally, a decrease in ATP levels was observed for BJ cells after 24 h treatment with phage vB_SauM-A at 10^9^ and 10^8^ PFU/mL (Figure 2). We also observed a statistically significant decrease in the viability of the BJ cells caused by the highest concentration of phage vB_SauM-D after 4 and 24 h of incubation (Figure 1). As well, a decrease in ATP levels in comparison with control was observed after 24 h of treatment with 10^9^ PFU/mL (Figure 2). Phage vB_SauM-C had no effect on cell viability measured either by neutral red uptake or ATP levels regardless of concentration used (Figure 1 and Figure 2).

### 2.2. Phage Influence on Cell Membrane Integrity of BJ Cells

**Figure 3:**
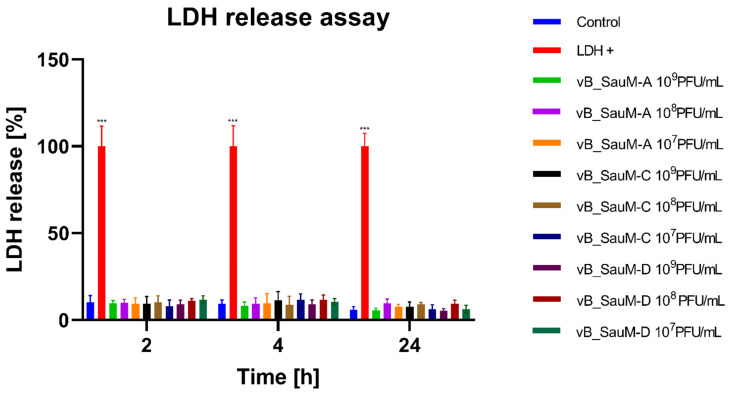
LDH release assay from BJ cells treated with phages vB_SauM-A, vB_SauM-C and vB_SauM-D at different concentrations after 2, 4 and 24 h of incubation in comparison to the untreated negative control (blue) and LDH release positive control (red). Mean values from three independent experiments are shown, with error bars representing SD. Statistical analysis was performed using the Kruskal–Wallis test followed by Dunn’s multiple comparison test for values with nonparametric distribution, with *** *p* < 0.001.

In order to analyze if the application of a high concentration of phage preparations could result in the disruption of cell membranes and cause cell death, we performed a colorimetric lactate dehydrogenase (LDH) release assay. We observed that the levels of released LDH for cells treated with phages were not significantly different from untreated negative control (Figure 3). Furthermore, Wilcoxon signed-rank test showed no significant differences in LDH levels between different incubation periods (Table S1).

### 2.3. Phage Activity against MDRSA Strains in Cell Line

**Figure 4:**
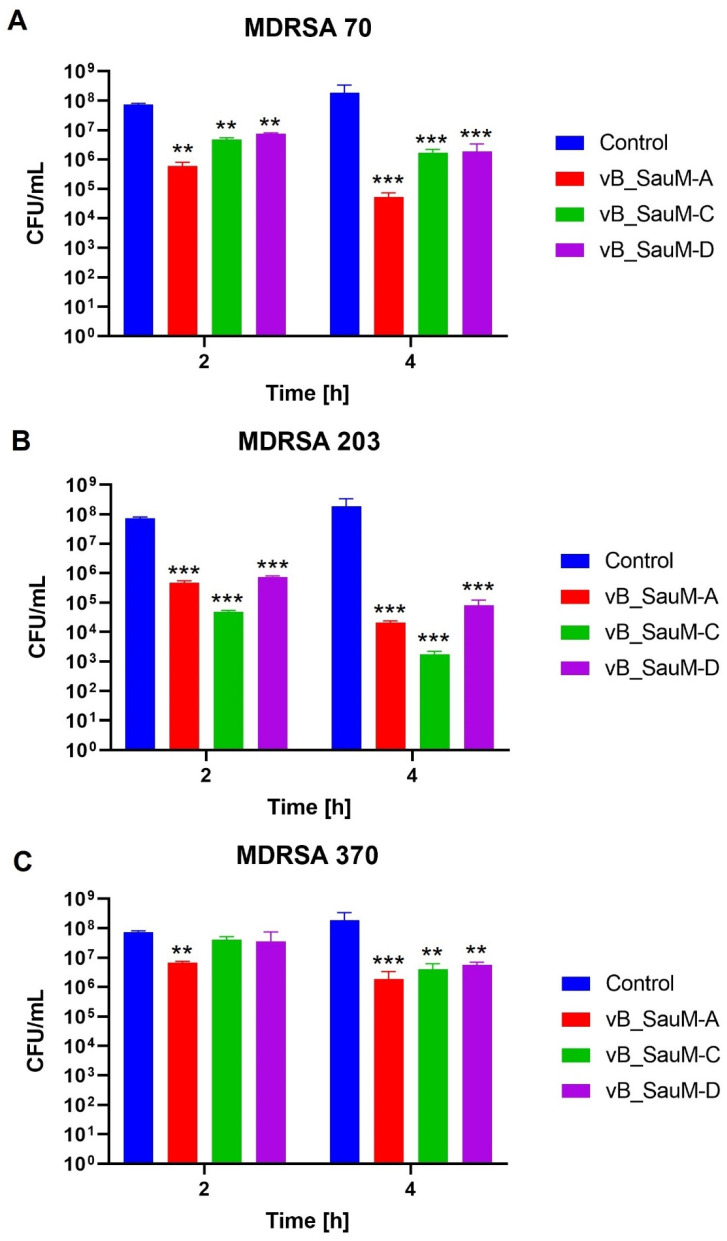
The CFU/mL of MDRSA strains 70 (**A**), 203 (**B**) and 370 (**C**) treated with phages vB_SauM-A, vB_SauM-C and vB_SauM-D at 10^7^ PFU/mL after 2 and 4 h of incubation in comparison to the negative control (non-treated with phages). Mean values from three independent experiments are shown, with error bars representing SD. Statistical analysis was performed using the Kruskal–Wallis test followed by Dunn’s multiple comparison test for values with nonparametric distribution, with ** *p* < 0.01 and *** *p* < 0.001.

**Figure 5:**
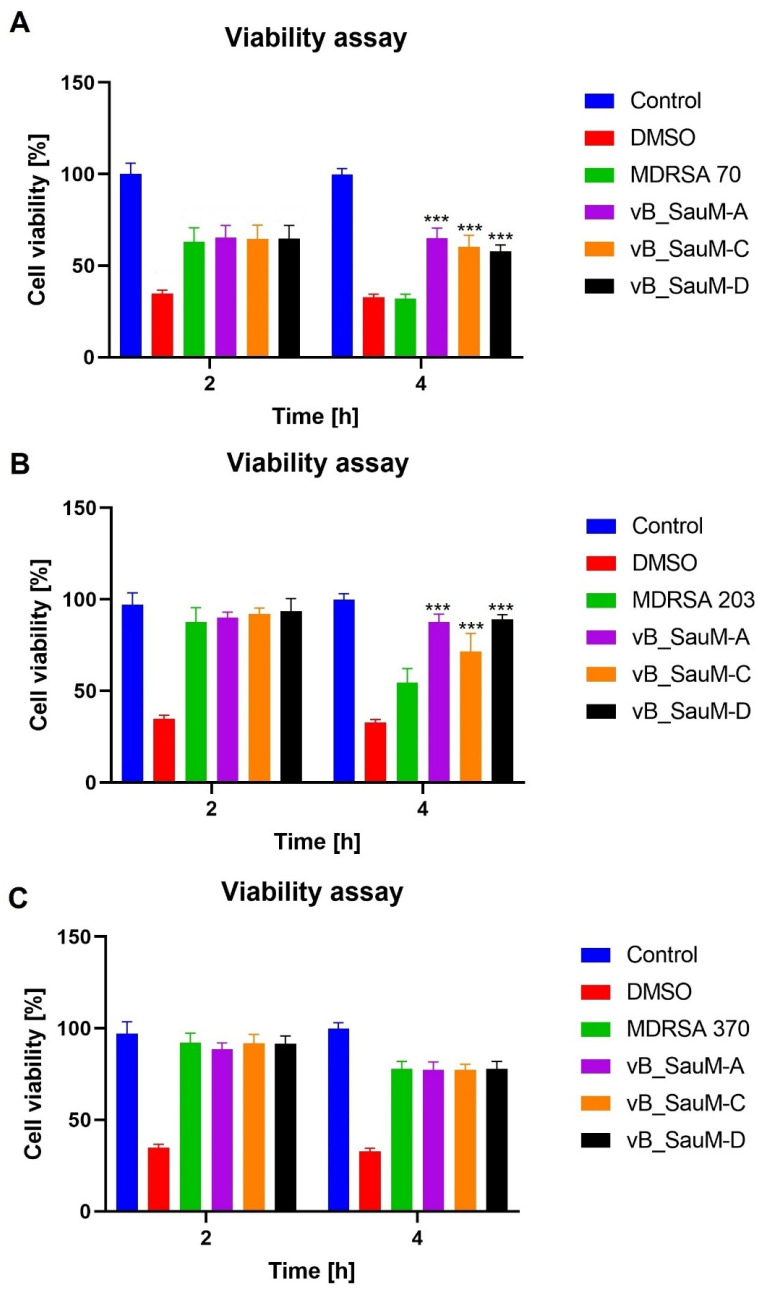
Cell viability of the BJ cells infected with MDRSA strains 70 (**A**), 203 (**B**) and 370 (**C**) treated with phages vB_SauM-A, vB_SauM-C and vB_SauM-D after 2 and 4 h of incubation in comparison to the phage-untreated control (green). Negative control (blue) and 10% DMSO-treated positive control (red) are visualized as referent values. Mean values from three independent experiments are shown, with error bars representing SD. Statistical analysis was performed using the Kruskal–Wallis test followed by Dunn’s multiple comparison test for values with nonparametric distribution, with *** *p* < 0.001.

To assess whether bacteriophages are able to reduce the number of bacteria on the surface of fibroblasts, we inoculated the BJ cell culture with one of three different MDRSA strains that were previously characterized: MDRSA 70, 203 and 370. These three strains have been identified as strong biofilm producers, with a possible toxic effect on human cells; therefore, it was assumed that they should successfully attach to BJ cells [20]. Two hours after the infection, bacteriophages were added to a final concentration of 10^7^ PFU/mL and bacterial titer was assessed after 2 and 4 h after the phage application. A titer of 10^7^ PFU/mL was chosen, as for all three phages this concentration did not influence either the viability or membrane integrity of the BJ cells. We observed that bacteriophages were able to reduce bacterial titer in the culture, especially after a 4 h incubation period where all three phages showed a statistically significant reduction of bacterial titer for all three MDRSA strains (Figure 4). The highest reduction in CFU/mL was observed for strains 70 and 203, which showed ~3 log reduction on average after a 4 h incubation period (Figure 5a,b). The phages were least effective against MDRSA strain 370, which showed only ~1.5 log reduction in CFU/mL (Figure 5c).

### 2.4. The Influence of Phage Lytic Activity against MRDSA Strains on Cell Viability and Membrane Integrity

**Figure 6:**
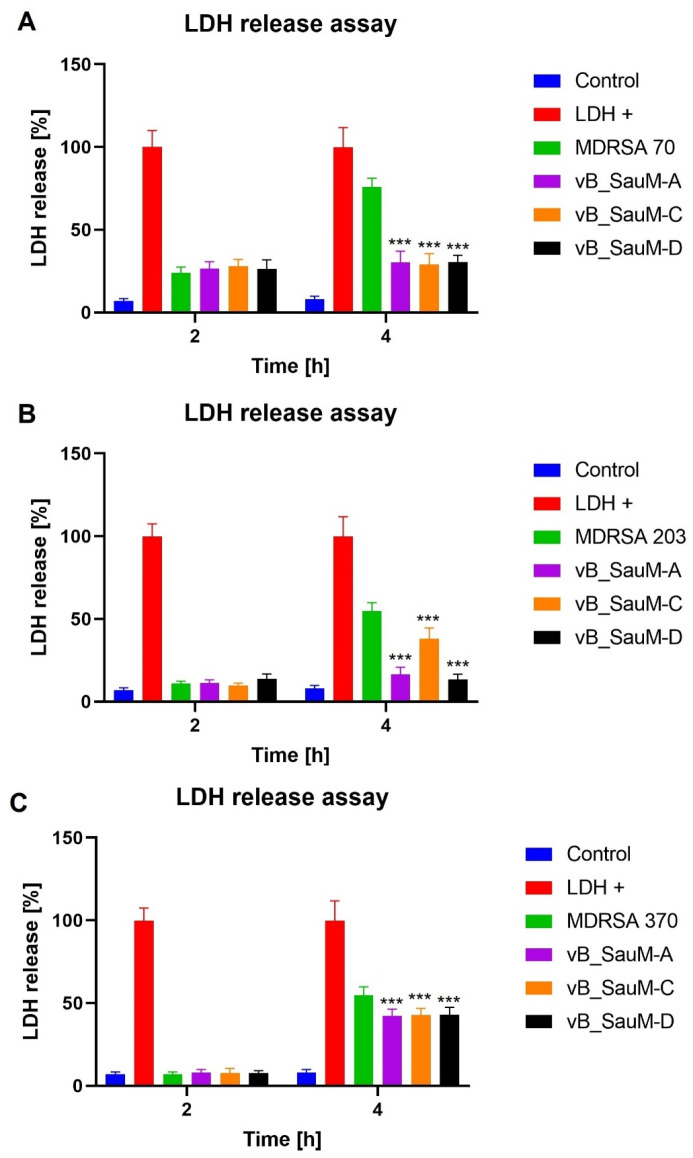
LDH release assay from BJ cells infected with MDRSA strains 70 (**A**), 203 (**B**) and 370 (**C**) treated with phages vB_SauM-A, vB_SauM-C and vB_SauM-D after 2 and 4 h of incubation in comparison to the phage-untreated control (green). Negative control (blue) and 10% DMSO-treated positive control (red) are visualized as referent values. Mean values from three independent experiments are shown, with error bars representing SD. Statistical analysis was performed using the Kruskal–Wallis test followed by Dunn’s multiple comparison test for values with nonparametric distribution, with *** *p* < 0.001.

*S. aureus* has been observed to have a negative effect on cell viability due to the vast production of different toxins [21,22]. Upon bacterial cell death, the toxins are no longer being produced. However, they remain in a culture and can still negatively influence cell condition. Furthermore, upon lysis of Gram-positive bacteria, compounds of bacterial cell wall such as lipoteichoic acid are released into the environment in large quantities. This has been shown to have a negative effect on eukaryotic cells [15]. Therefore, in addition to phage efficacy analysis in the reduction of MDRSA titer in fibroblast culture, we decided to analyze the influence of phage lytic activity towards bacteria on fibroblast cell viability and membrane integrity. We compared phage-treated samples with infected, untreated ones as well as with untreated negative control and positive control treated with DMSO (for NR stain) or lysis buffer (for LDH release assay). We observed that, for MDRSA strain 70, the treatment with all three phages resulted in the inhibition of the toxic effect on the BJ cells after 4 h of incubation (Figure 5a and Figure 6a). The negative effect of strains 203 and 370 was not observed after the first 2 h of incubation. However, 4 h incubation resulted in a significant decrease in viability (to ~55% for MDRSA 203 and ~80% for MDRSA 370) (Figure 5b,c). The treatment with all three phages did not inhibit the negative influence of MDRSA 370 on the BJ cells. However, the levels of released LDH were lower when compared with the untreated infected sample (~45% in comparison to ~58%). For MDRSA 203, the observed results depended on the phage used as treatment, with phages vB_SauM-A and vB_SauM-D resulting in the inhibition of the decrease in cell viability from bacterial presence. Treatment with phage vB_SauM-C resulted in a smaller reduction of cell viability and lower levels of released LDH in comparison to the infected, phages-untreated sample. However, the overall condition of the BJ cells still deteriorated between 2 and 4 h of the incubation period (Figure 5b and Figure 6b).

## 3. Discussion

MDRSA is a leading cause of nosocomial skin and soft tissue infections [9]. In recent years, it has also been reported to be isolated from patients with skin infections that have never been hospitalized before [12]. Phage therapy is seen as a promising way of treatment for multi-drug-resistant bacteria, including MDRSA [23,24,25]. Queen Astrid Military Hospital (Belgium) and the Phage Therapy Unit of Hirszfeld Institute of Immunology and Experimental Therapy (Poland) are leading facilities in applying phage therapy as a treatment for patients with skin and soft tissue infections caused by multidrug-resistant bacteria [26,27,28]. There are also other reports of effective applications of phage preparations to treat skin and wound infections [13,29]. The use of phage-based therapeutics to combat superficial bacterial infections is, by some, seen as being the easiest in the application and bearing the least possibility of adverse side effects [14,30]. However, since phages are able to interact with eukaryotic cells, it is important to analyze whether they can influence the condition and viability of cells [3]. The interactions between phages and those cells seem to be dependent on the type and properties of the cell [1,4,5]. This adds to the argument for phage preparations have the greatest potential to be used in the treatment of superficial infections. However, it is crucial to carefully analyze the properties and cytotoxicity of each phage showing therapeutic potential before it could be applied as a treatment [31].

In our study, we have analyzed how three staphylococcal phages, vB_SauM-A, vB_SauM-C and vB_SauM-D, affect the metabolic state of human fibroblasts to assess their potential for therapeutic use [18]. The phages have been shown to be active against MDRSA strains isolated from hospitalized patients, including those with skin and soft tissue infections, using both in vitro and in vivo models [18,19]. We have observed that treatment with high concentrations (10^9^ PFU/mL) of two phages, vB_SauM-A and vB_SauM-D, resulted in a slight but statistically significant reduction in viability and metabolic activity of human skin fibroblasts. When human fibroblasts were treated with the vB_SauM-C phage, no differences in LDH levels or metabolic activity were observed when compared to the untreated control. Therefore, it could be assumed that the vB_SauM-C phage did not interact with BJ cells to the same extent as the vB_SauM-A and vB_SauM-D phages. It is worth to mention that the reduction in cells’ viability was observed only when a high concentration (10^9^ or 10^8^ PFU/mL) of phages was used, while the concentration of 10^7^ PFU/mL did not affect the metabolic activity and viability of the cells. The research performed by other groups reports that the use of phages at doses as low as 10^7^ PFU/mL or 10^6^ PFU/mL was effective in reducing the number of *S. aureus* in skin infections in a murine model [32,33,34,35]. The case studies where phages were used as experimental or compassionate treatment have also reported success even when low doses were applied [13,26,36]. Therefore, it is possible to obtain the desired therapeutic effect without the possible negative influence of bacteriophages on cells [25,30]. This is further supported by the observation that the addition of 10^7^ PFU/mL vB_SauM-A, vB_SauM-C or vB_SauM-D to MDRSA-infected fibroblast culture resulted in a statistically significant reduction of bacterial CFU/mL, especially in the cases of strains MDRSA 70 and 203, where bacterial titer reduction was comparable with the results obtained using a pure culture staphylococcal biofilm model [19]. Similarly, high phage lytic activity against *S. aureus* and other pathogens in cell culture was observed and reported by other research groups [3,37]. Shan et al., observed an increase in phage lytic activity towards *C. difficile* in HT-29-infected cell culture in comparison to experiments performed with bacterial biofilm [38]. This suggests a potential “cooperation” between phages and eukaryotic cells that leads to increased antibacterial effects [3,39]. However, even though the phage lytic activity against pathogens in cell culture becomes a subject of interest to more researchers, there is still little data on how this activity influences eukaryotic cells. The release of virulence factors and cell debris as a result of phage-induced lysis could potentially lead to increased inflammation and apoptosis [3,16]. This has been reported to happen in the case of the use of some antibiotic groups, especially β-lactams that target cell wall synthesis [40,41,42]. Therefore, it is important to study the influence of phage lytic activity against pathogens on cell health to assess the risk of potential side effects. In our study, we observed that the phages could inhibit the toxic effect of MDRSA strains on BJ. However, this effect was both phage- and strain-dependent as the treatment with all three phages inhibited LDH release and cell viability decline for MDRSA strain 70. The effects of strain 203 were mixed and varied depending on the phage used, with phages vB_SauM-A and vB_SauM-D being the most effective. The toxic effect of strain 370 was reduced but not inhibited. However, this study showed that phage-induced bacterial lysis did not result in a rapid decline in human cell condition. Thus, vB_SauM-A, vB_SauM-C or vB_SauM-D phages could be used to treat staphylococcal skin infections in the future with minimal risk of side effects. However, in vivo mammalian models should be used for verification.

## 4. Materials and Methods

### 4.1. Human Fibroblast Cell Culture

Normal human fibroblasts, BJ line (CRL-2522™), Eagle’s Minimum Essential Medium (EMEM) medium and fetal bovine serum (FBS) were obtained from ATCC, Manassas, VA, USA. Cells were grown in accordance with ATCC guidelines in EMEM medium supplemented with 10% fetal bovine serum (FBS) at 37 °C in a humidified atmosphere of 95% air and 5% CO_2_ in the HeraCell 150 incubator (Heraeus, Hanau, Germany).

### 4.2. Bacteriophages

Phages vB_SauM-A, vB_SauM-C and vB_SauM-D have been characterized previously [18,19]. For phage purification, polyethylene glycol (PEG) 8000 (BioShop, Burlington, ON, Canada) was added to phage lysate (final concentration 10% *w/v*) and stirred overnight at 4 °C. The precipitate was collected by centrifugation at 11,000× *g* for 20 min at 4 °C and suspended in EMEM medium. PEG8000 was removed by adding the same volume of chloroform and centrifugation at 3000× *g* for 15 min. After the PEG 8000 was removed, FBS was added to the medium to a final concentration of 10%. Purified lysates were stored at 4 °C [18].

### 4.3. MDRSA Clinical Strains

MDRSA strains 70, 203 and 370 from the collection of the Department of Medical Microbiology have been chosen for this study. The strains have been previously characterized in terms of antibiotic resistance, production of toxins and biofilm formation [18,20]. Lytic activity of phages vB_SauM-A, vB_SauM-C and vB_SauM-D have also been assessed and described as high against all three strains [18,19]. The description of the strains is presented in Table 1. The strains were chosen for the study based on their biofilm formation abilities (strong biofilm producers) [20], the efficacy of plating of all three phages [18] and phage activity against biofilm formed by those strains and in vivo experimental results [19].

### 4.4. Neutral Red Cytotoxicity Assay

The assay was performed in accordance with the protocol published previously [43]. In brief: BJ cells were plated into 96-well tissue culture plates (Nest Scientific Biotechnology, Wuxi, China) at a density of 6 × 10^3^ per well and allowed to attach for 24 h in EMEM supplemented with 10% FBS. After 24 h, the medium was removed, and 10% dimethyl sulfoxide (DMSO) (Sigma-Aldrich, St. Louis, MO, USA)-treated cells were used as a positive control, while non-treated cells were considered a negative control. Cells were treated with phages vB_SauM-A, vB_SauM-C or vB_SauM-D at concentrations 10^9^, 10^8^ and 10^7^ PFU/mL (Plaque-Forming Unit/mL) suspended in EMEM medium supplemented with 10% FBS. Cells were incubated for 2, 4 and 24 h at 37 °C in a humidified atmosphere of 95% and air/5% CO^2^. Following incubation, the supernatants were discarded and replaced with 100 µL of non-supplemented EMEM containing 0.33% neutral red (Sigma-Aldrich) diluted 1:40. After 2 h incubation at 37 °C, the neutral red medium was removed, and cells were washed with 100 µL of Phosphate-Buffered Saline (PBS) per well. Cells were then treated with 150 µL of a solution containing 50% ethanol (Alchem, Torun, Poland), 49% distilled H_2_O and 1% acetic acid (Alchem, Torun, Poland) and incubated with shaking at 37 °C for 10 min in order to extract the dye into solution. Absorbance was measured at 540 nm (SynergyH1, BioTek Instruments, Winooski, VT, USA).

### 4.5. ATP Assay

The assay was performed using CellTiter-Glo 2.0^®^ (Promega, Madison, WI, USA) in accordance with the manufacturer’s protocol [44]. In brief: BJ cells were plated and treated with bacteriophages as described earlier in this section (see Section 4.3). The plates were then incubated at a benchtop for 30 min to equilibrate the plate and its contents to room temperature. An amount of 100 µL of CellTiter-Glo 2.0^®^ reagent was added to each well, mixed for 5 min on an orbital shaker and incubated at room temperature for another 10 min. The luminescence was measured using Synergy H1 plate reader (BioTek Instruments, Winooski, VT, USA).

### 4.6. Lactate Dehydrogenase (LDH) Release Assay

The LDH release assay was performed using CytoTox 96^®^ (Promega, Madison, WI, USA) in accordance with the manufacturer’s protocol. In brief: BJ cells were plated and treated with bacteriophages as described earlier in this section (see Section 4.3). Forty-five minutes before the end of the incubation period, 10 µL of 10 × Lysis Buffer was added to the wells, which served as a positive control. After 45 min, 50 µL of the medium was transferred onto a fresh microplate and 50 µL of LDH Substrate Mix was added to each well. The plate was then incubated in the dark for 30 min. After the incubation period, 50 µL of Stop Solution was added and the absorbance was immediately measured at 490 nm [45].

### 4.7. Activity of Phages in MDRSA-Infected Fibroblast Culture

MDRSA strains were grown in LB (Luria-Bertani) medium (BioShop, Burlington, ON, Canada) as follows: an overnight culture of the strain was added to fresh medium at 1:100 ratio and then incubated with shaking at 37 °C and 120 rpm until the optical density of OD_600_ = 0.1. One mL of culture was then centrifugated for 5 min at 4 °C and 1000× *g*. The supernatant was discarded and the pellet was washed twice with 0.9% NaCl in order to dispose of the medium and bacterial metabolites. Bacteria were then suspended in 1 mL of EMEM medium and used immediately in cell culture.

BJ cells were plated into 96-well tissue culture plates (Nest Scientific Biotechnology, Wuxi, China) at a density of 6 × 10^3^ per well and allowed to attach for 24 h in EMEM supplemented with 10% FBS. After 24 h, the medium was removed, and 10% DMSO (Sigma-Aldrich, St. Louis, MO, USA)-treated cells were used as a positive control, while non-treated cells were considered a negative control. Cells were infected with 10^6^ CFU/mL (Colony-Forming Unit/mL) of MDRSA strains 70, 203 or 370. After 2 h of incubation bacteriophages were added to 10^7^ PFU/mL. One set of wells was left untreated as a control. After 2 and 4 h of incubation, 50 µL of the medium was removed and serial dilutions were made in 0.9% NaCl. Fifty µL of the each dilution was spread on LB-agar plates. The samples were then incubated overnight at 37 °C and then for colonies for CFU/mL count [38].

### 4.8. The Influence of Bacteriophage Lytic Activity on Fibroblasts’ Viability

The cells were treated as described above (see Section 4.6). After the incubation period, 50 µL of the medium was removed for LDH levels assay (see Section 4.5) while the cells were stained with neutral red in accordance with the protocol described above (see Section 4.3) [38].

### 4.9. Statistical Analysis

All experiments were carried out in three biological replicates. Technical replicates were averaged to produce replicate means that were used for analysis. Mean values were compared using the Kruskal–Wallis test followed by Dunn’s multiple comparison test for values with the nonparametric distribution. In order to assess if there is a significant difference between time points, the Wilcoxon signed-rank test was used. Differences were considered statistically significant if *p* < 0.05.

## 5. Conclusions

Our work demonstrated that all three tested phages, vB_SauM-A, vB_SauM-C and vB_SauM-D, were able to effectively reduce the number of MDRSA on the surface of BJ cells, and thus reduce the cytotoxicity of the bacteria on fibroblast cells. However, we observed that high concentrations (10^9^ PFU/mL) of vB_SauM-A and vB_SauM-D phages may have a negative effect on BJ cell viability. Our research suggests that the three phages studied have the potential to be used as therapeutic agents against MDRSA-induced skin infections. However, dosing needs to be more carefully evaluated to reduce the risk of potential side effects.

## Figures and Tables

**Table 1:** Antibiotic resistance and efficacy of plating of phages vB_SauM-A, vB_SauM-C and vB_SauM-D of MDRSA strains 70, 203 and 370.

MDRSA Strain	Antibiotic Resistance ^1^	Efficacy of Plating of a Phage vB_Sau-M ^2^		
		A	C	D
70	Fox P E CC NOR CIP TE	++	+++	+++
203	Fox P E CC NOR CIP	+++	+++	+++
370	Fox P E CC NOR CIP	++	+++	+++

^1^ Antibiotics abbreviations: FOX, cefoxitin (30 g); P, penicillin (10 U); E, erythromycin (15 g); CC, clindamycin (2 g); NOR, norfloxacin (10 g); CIP, ciprofloxacin (5 g); ^2^ vB_SauM-A; vB_SauM-C; vB_SauM-D; +++ phage titer ≥10^9^ PFU/mL, ++ phage titer 10^8^–10^6^ PFU/mL. Source: Łubowska et al., 2019 [18], modified.

## Data Availability

Raw data are available from the authors upon request.

## Supplementary Materials

The following supporting information can be downloaded at: https://www.mdpi.com/article/10.3390/ijms24065961/s1.
