# Supplementary material for: The Influence of Bacteriophages on the Metabolic Condition of Human Fibroblasts in Light of the Safety of Phage Therapy in Staphylococcal Skin Infections
Source: Int J Mol Sci. 2023 Mar 22;24(6):5961. doi: 10.3390/ijms24065961 (PMC10055722; doi:10.3390/ijms24065961)
Supplement: Supplementary file 1 [file ijms-24-05961-s001.zip › ijms-2283375-supplementary.pdf]

Table S1. Results of the Wilcoxon signed-rank test for the change LDH release levels over time. Statistical analysis was performed using GraphPad Prism 9.

	Control	LDH +	vB_SauM-A 10^9 PFU/ml	vB_SauM-A 10^8 PFU/ml	vB_SauM-A 10^7 PFU/ml	vB_SauM-C 10^9 PFU/ml	vB_SauM-C 10^8 PFU/ml	vB_SauM-C 10^7 PFU/ml	vB_SauM-D 10^9 PFU/ml	vB_SauM-D 10^8 PFU/ml	vB_SauM-D 10^7 PFU/ml
Theoretical median	0	0	0	0	0	0	0	0	0	0	0
Actual median	9,361	100	8,172	9,509	9,312	9,438	9,054	7,916	9,008	11,15	10,52
Number of values	3	3	3	3	3	3	3	3	3	3	3
Wilcoxon Signed Rank Test											
Sum of signed ranks (W)	6	6	6	6	6	6	6	6	6	6	6
Sum of positive ranks	6	6	6	6	6	6	6	6	6	6	6
Sum of negative ranks	0	0	0	0	0	0	0	0	0	0	0
P value (two tailed)	0,25	0,25	0,25	0,25	0,25	0,25	0,25	0,25	0,25	0,25	0,25
Exact or estimate?	Exact	Exact	Exact	Exact	Exact	Exact	Exact	Exact	Exact	Exact	Exact
P value summary	ns	ns	ns	ns	ns	ns	ns	ns	ns	ns	ns
Significant (alpha=0.05)?	No	No	No	No	No	No	No	No	No	No	No
Discrepancy	9,361	100	8,172	9,509	9,312	9,438	9,054	7,916	9,008	11,15	10,52
